# Selected pharmacokinetic issues of the use of antiepileptic drugs and parenteral nutrition in critically ill patients

**DOI:** 10.1186/1475-2891-9-71

**Published:** 2010-12-31

**Authors:** Muhannad RM Salih, Mohd Baidi Bahari, Arwa Y Abd

**Affiliations:** 1 Department of Clinical Pharmacy, School of Pharmaceutical Sciences, Universiti Sains Malaysia, 11800 Penang, Malaysia

## Abstract

**Objectives:**

To conduct a systematic review for the evidence supporting or disproving the reality of parenteral nutrition- antiepileptic drugs interaction, especially with respect to the plasma protein-binding of the drug.

**Methods:**

The articles related to the topic were identified through Medline and PubMed search (1968-Feburary 2010) for English language on the interaction between parenteral nutrition and antiepileptic drugs; the search terms used were anti-epileptic drugs, parenteral nutrition, and/or interaction, and/or in vitro. The search looked for prospective randomized and nonrandomized controlled studies; prospective nonrandomized uncontrolled studies; retrospective studies; case reports; and in vitro studies. Full text of the articles were then traced from the Universiti Sains Malaysia (USM) library subscribed databases, including Wiley-Blackwell Library, Cochrane Library, EBSCOHost, OVID, ScienceDirect, SAGE Premier, Scopus, SpringerLINK, and Wiley InterScience. The articles from journals not listed by USM library were traced through inter library loan.

**Results:**

There were interactions between parenteral nutrition and drugs, including antiepileptics. Several guidelines were designed for the management of illnesses such as traumatic brain injuries or cancer patients, involving the use of parenteral nutrition and antiepileptics. Moreover, many studies demonstrated the in vitro and in vivo parenteral nutrition -drugs interactions, especially with antiepileptics.

**Conclusions:**

There was no evidence supporting the existence of parenteral nutrition-antiepileptic drugs interaction. The issue has not been studied in formal researches, but several case reports and anecdotes demonstrate this drug-nutrition interaction. However, alteration in the drug-free fraction result from parenteral nutrition-drug (i.e. antiepileptics) interactions may necessitate scrupulous reassessment of drug dosages in patients receiving these therapies. This reassessment may be particularly imperative in certain clinical situations characterized by hypoalbuminemia (e.g., burn patients).

## Introduction

In the past, many efforts have been made to supply nutrients intravenously through cannulation of peripheral veins. However, administration of different feeding solutions like milk, salted water and glucose were associated with thrombophlebitis or fluid overload [1]. In 1968, Dudrick and his colleagues administered a nutrition solution through the superior vena cava, enabling the administration of a small volume of nutrition solution with high nutrient concentrations [2]. The practice of intravenous feeding was then utilised to nourish an infant for more than 6 weeks. Subsequently, many adult and paediatric patients with gastrointestinal abnormalities were sustained by nutrition support via a central venous catheter [3,4]. A great advancement has been made in methods for IV cannulation and IV nutrient liquids formulation during the past four decades. Currently, the IV administration of complex nutrient mixture is equated with parenteral nutrition (PN) and has become a vital part of the health management for both outpatients and hospitalised patients who are not able to eat or consume nutrients through the gastrointestinal tract [1].

The parenteral nutrition technique can be applied to all patients regardless of their gastrointestinal function or metabolic status. However, many challenges and controversial issues may be involved in the assessment of beneficial effects gained from IV nutrition in certain patients [5-9]. Malnutrition has been found to have negative effects on the incidence of postoperative complications, length of hospitalisation, and mortality [10-20]. A patient's nutritional status can be improved and reinstated by both parenteral and enteral nutrition [21-32]. Although previous studies have demonstrated a positive effect of nutrition support on length of hospital stay, morbidity, and mortality [8,18,28,33-36]. A meta-analysis study has revealed that total parenteral nutrition does not have an effect on the death rate of surgical patients, but it may possibly decrease the complication rate, particularly in malnourished patients [37]. Consequently, clinicians must consider the general guidelines and clinical judgments as the basis and structure for deciding the suitability of nutrition support.

Several questions can be considered in deciding whether nutrition support is clinically beneficial for a given patient [38]:

• Does malnutrition exist?

• Will the malnutrition contribute to an increased likelihood of mortality or morbidity, or decrease in the response to other therapies?

• Is the existing malnutrition a consequence of starvation, altered metabolism, or a combination of both?

• Will treating the malnutrition with either enteral or parenteral nutrition result in an improvement in nutritional status?

• Will an improvement in nutritional status affect morbidity, response to therapy, or mortality?

• Considering the effect of nutrition support on patient outcome, will the cost of providing nutrition support outweigh the cost of not providing nutrition support?

A number of aspects have to be considered when assessing a patient's suitability for nutrition support, including the patient's age, the existence of critical illness, the occurrence of malnutrition and its severity, and the importance of nutrition support to the whole therapeutic management plan [39]. The cornerstone that governs the decision on which route of nutrition support should be utilised in a certain patient is the functional capability of the gastrointestinal tract. Generally, the enteral route is more favoured than the PN as it consistently meets the nutrition requirements [40]. However, PN should be used when nutrition requirements cannot be met by enteral nutrition and when the gastrointestinal tract is not functioning or cannot be accessed. As with any therapy, patients should be monitored and should undergo thorough routine evaluations to assess the best route of nutrition support as frequently as possible.

Many types of complications may be associated with the use of PN, such as mechanical and septic complications related to intravenous catheters. These complications may be observed directly after placing of a central venous catheter and may include intravascular and extravascular malposition, hydrothorax, hemothorax, brachial nerve plexus injury and pneumothorax [41-45]. Thrombosis is considered to be one of the late complications that may happen in the lumen of the intravenous catheter and may also affect the blood vessel around the catheter [41-47]. Contaminated IV fluid or contamination of the catheter site may lead to septicaemia, which is considered to be a serious, life-threatening complication [41-46,48-55]. In addition to the mechanical and septic complications, various sorts of metabolic complications may be associated with PN. The most frequently reported abnormalities are hypokalaemia, hypomagnesaemia, hypophosphataemia and hyperglycaemia. Routine monitoring of these serum electrolytes is very important to identify any electrolyte disturbances and to avoid any possible complications [56].

Improvement of pharmaceutical safety has been studied in terms of stability and compatibility for total parenteral nutrition (TPN) admixture [57]. Parenteral drugs should be given separately from TPN solutions. However, sometimes it is impossible to absolutely dedicate the lumen to administration of TPN, particularly in ICU and cancer patients. In such patients, the number of IV drugs sometimes exceeds the number of available access sites [58]. Although intravenous drug-drug incompatibility has been widely discussed [59], information relating to drug-PN incompatibility is limited. The problem is compounded by the huge variability in nutrition components and concentrations; furthermore, there are different ranges and concentrations of drugs that may be used [58]. Even though PN plays a significant role in a variety of cases, there are some potential interactions that might occur with drugs administered concurrently with PN. Therefore, the goal of this article was to perform a systematic review for the evidence supporting or disproving the existence of PN- antiepileptic drugs interaction, especially with respect to the plasma protein-binding of the drug.

The authors conducted Medline and PubMed search from 1968 to February 2010 for English- language articles describing the interaction between PN and antiepileptic drugs; the search terms used were anti-epileptic drugs, PN, and/or interaction, and/or in vitro. The search looked for prospective randomized and nonrandomized controlled studies; prospective nonrandomized uncontrolled studies; retrospective studies; case reports; and in vitro studies. Full text of the articles were then traced from the Universiti Sains Malaysia (USM) library subscribed databases, including Wiley-Blackwell Library, Cochrane Library, EBSCOHost, OVID, ScienceDirect, SAGE Premier, Scopus, SpringerLINK, and Wiley InterScience. The articles from journals not listed by USM library were traced through inter library loan.

### Study designs

After performing the required search, a huge lacking of the formal research evidence was found. So that, this review will mostly depend on the use of anecdotes and case reports to describe some of the pharmacokinetic issues (mainly plasma protein binding) of the use anti-epileptic drugs and PN in critically ill patients.

### Parenteral nutrition and anticonvulsants in traumatic brain injury

Serious head injury is often followed by hypermetabolism, hypercatabolism, and nitrogen loss [60,61]. Hypermetabolism is characterised by an increase in the levels of catabolic hormones, especially cortisol, epinephrine, norepinephrine, and glucagon. Hypermetabolism results from an elevation in the level of catabolic hormones, cytokines, and catecholamines, leading to increases in the cardiac output, tachycardia, and mild hypertension. Consequently, oxygen consumption and caloric requirement will be increased. A hypercatabolic state is the consequence of amino acid mobilisation from skeletal muscles, gluconeogenesis, increased nitrogen excretion, loss of weight, and muscle wasting. In addition to the skeletal muscles, visceral and circulating protein will undergo proteolysis if aggressive nutritional support is not provided [60,62]. Hypermetabolic and hypercatabolic states can precipitate a condition of severe malnutrition that leads to several complications, such as poor wound healing, loss of body mass, immunosuppression, infection, and multiple organ failure [60,63]. Therefore, nutritional support is mainly given to ensure the required calories and proteins and to achieve a positive nitrogen balance [63]. However, a positive nitrogen balance may not be reached for 2 to 3 weeks after injury, even with aggressive nutritional support [60,64]. Nutritional therapy is recommended by the Guidelines for the Management of Severe Head Injury, which state that nutrition support should start within 72 hours of injury and with complete caloric replacement should be achieved within 7 days [65].

Total parenteral nutrition provides early feeding if enteral nutrition is not accessible or enteral feeding is not feasible due to gastrointestinal malfunction. Total parenteral nutrition provides a steadier nutritional intake and causes less diarrhoea than enteral nutrition [63].

Seizure is one of the most common posttraumatic complications in patients with a recent head or brain injury [66]. Jennett confirmed that the severity of a brain injury can be used to estimate the probability of early (within 7 days of injury) and late (after 7 days) risk of seizures [67]. Annegers and colleagues have demonstrated that a small increase in the risk of epilepsy may be noted in subsequent years with mild (loss of consciousness or amnesia for less than 30 minutes) and moderate brain injuries (loss of consciousness, amnesia for 30 minutes to 24 hours, or skull fracture). In addition, a significantly increased risk of epilepsy is associated with more severe brain injuries (loss of consciousness, amnesia lasting for more than 24 hours, subdural haematoma, or brain contusion) over the next 10 years [68]. A systematic review of ten randomised controlled trials revealed consistent evidence regarding the early management with anticonvulsant drugs after head injury. Phenytoin is the gold standard; however, phenobarbital and carbamazepine also reduced the relative risk of early seizures [69]. Moreover, valproic acid was suggested as an alternative agent for posttraumatic seizure prophylaxis; however, patients receiving valproic acid had a slightly higher mortality rate than those receiving other antiepileptic drugs [70].

### Parenteral nutrition and anticonvulsants in cancer patients

Many review articles and meta-analysis studies have been published to evaluate the effects of nutritional support in cancer patients. In this sequence, a consensus has been made to create standards for nutritional support in cancer patients [71]. Klein et al. utilised 28 randomised controlled trials to evaluate the use of PN in cancer patients. They concluded that the use of PN in patients with cancer of the gastrointestinal tract is justified. Although the risk of infection increased in patients receiving both PN and chemotherapy, the authors did not rule out the potential benefit of PN in cancer patients treated with radiotherapy and chemotherapy [72]. Another review article including 12 randomised controlled trials was published by the American College of Physicians. The aim of this review was to predict the effect of PN on survival and tumour response rate in patients undergoing chemotherapy. The findings of the pool analysis revealed that the tumour response rate was not improved by PN and that there was no significant difference between the control patients and those who received PN in terms of the survival rate [73]. The American Society for Parenteral and Enteral Nutrition (ASPEN) recommended the following practice guidelines [74]:

• Enteral and parenteral nutrition may have positive effects in some severely malnourished cancer patients or those who are unable to take adequate oral nutrition for more than one week. Patients under this category should be given nutrition support, if possible, in concurrence with the start of oncologic therapy.

• Nutritional support is not routinely recommended for mildly malnourished or well-nourished patients and those who are able to receive adequate nutrition via oral intake, even if they are undergoing chemotherapy, radiotherapy, or surgery.

• Total parenteral nutrition may have an invaluable effect in the management of patients with advanced cancer that are identified as unresponsive to radiation therapy or chemotherapy.

Furthermore, a prospective evaluation of quality of life in one hundred forty-six patients with head and neck cancer was reported [75]. The results revealed that nutrition support should be applied in cancer patient because its positive effects can improve the overall quality of life. In addition to nutritional support, anticonvulsant drugs may be incorporated in the treatment of cancer patients because seizure is a common neurological complication in patients with primary brain tumours and brain metastases [76,77]. A large discrepancy in seizure frequency was noted between patients with different primary brain tumours and patients with cerebral metastases from various primary tumours (Table 1) [77].

**Table 1:** Seizure frequency within different primary brain tumours and within cerebral metastases from various primary tumours

Cerebral tumour	Frequency
**Primary brain tumours**	

Malignant Glioma	50%

Low Grade Glioma	75%

Dysembryoblastic neuroepithelial	100%

Ganglioglioma	90%

PCNSL	10%

**Cerebral metastases**	

Melanoma	67%

Lung	39%

Unknown histology	25%

Gastrointestinal tumours	21%

Breast cancer	16%

Non Hodgkin Lymphoma	15%

Gynaecological	11%

Prostate cancer	0%

Others	12%

Total	20-40%

**Other neurooncological disease**	

Neoplastic Meningitis	10%

Oncological patients in general	14%

From Oberndorfer and Grisold (2008) [77] with permission of the author and the publisher.

The majority of brain tumour patients need chronic treatment with antiepileptic drugs because seizures are common in this population of patients. Sometimes, prophylactic use and acute treatment with antiepileptic drugs might be necessary [77]. Actually, clinicians follow general guidelines for the acute therapeutic management of seizures in patients with both epilepsy and brain tumours, including intravenous benzodiazepines, valproic acid, and levetiracetam, followed by phenytoin and then barbiturate if necessary [78]. Older generations of antiepileptic drugs (phenytoin, carbamazepine, and oxcarbazepine) are hepatically metabolised and induce cytochrome P450, which can reduce the efficacy of chemotherapeutic agents. New generations of antiepileptic drugs might be more convenient for the chronic treatment of seizures in cancer patients in relation to the induction of liver enzyme [77].

Valproic acid is a broad-spectrum antiepileptic drug that has been considered as a safe and potent drug for many decades. Recently, many studies have shown that valproic acid has some anticancer properties [79-81]. However, further studies are required to evaluate the efficacy and safety of valproic acid; not only as an antiepileptic drug, but also as a potential anticancer drug in patients with brain tumours [77]. Although prophylactic administration of anticonvulsant drugs is evidence-based in some circumstances, it is not recommended and might not be effective at preventing the first seizure [76,77].

### Systematic categorisation of drug-nutrient interactions

Interactions between drugs and nutrients frequently occur and might have a negative impact on the patient's clinical outcome [82]. These interactions became more relevant with the dramatic development of nutrition support and with the increased use of multiple medications. Identification of clinically important interactions can help in avoiding or early management of adverse effects resulting from drug-nutrient interactions. This point was emphasised by the Joint Commission on Accreditation of Healthcare Organizations (JCAHO) through its standards that encourage clinicians to monitor any potential interactions between drugs and nutrients [83]. Based on the current information required for recognition and suitable management of drug-nutrient interactions in patients receiving parenteral and enteral nutrition, drug-nutrient interactions were sorted into four types [84]:

• *Ex vivo *biopharmaceutical inactivations

• Interactions affecting the absorption phase

• Interactions affecting systemic/physiologic dispositions

• Interactions affecting elimination/clearance

### *Ex vivo *biopharmaceutical inactivations

The contact between drug molecules and nutritional constituents that leads to physical or biochemical reactions may occur through *ex vivo *biopharmaceutical interactions. These interactions typically happen outside the body, either in the delivery system (e.g., infusion bags or infusion tubings) or during the compounding process. Several examples are available for this type of interaction, such as the physical incompatibility between PN fluid and intravenous drugs, the hydrolysis reaction that results from the direct mixing of oral liquid drugs with enteral feeding formula, and the destruction of fat emulsion that occurs when a large quantity of heparin is added into 3-in-1 parenteral nutrition formula. Crystals, precipitates or other undissolvable intermediates may result from these interactions as a final byproduct. Life-threatening events (e.g., occlusion of blood vessels) may occur from the systemic exposure of the patient to these byproducts. On the other hand, physical incompatibility with enteral nutrition usually does not cause life-threatening conditions, but it may result in a decrease in the therapeutic effect or poor nutritional improvement [84]. One of the common examples that can be seen is the dramatic reduction in the phenytoin absorption when given with enteral nutrition [85,86].

### Absorption interactions

Absorption interactions can occur with drugs and nutrients that are only taken or delivered orally or through enteral feeding delivery systems. The oral bioavailability of the active drug may increase or decrease as a result of these interactions [84]. Absorption interactions can be subclassified into the following three types: 1) presystemic metabolism (e.g., grape fruit juice with carbamazepine) [87], 2) presystemic transport (e.g., vitamin E and cyclosporine) [88], and 3) presystemic binding/complexation (e.g., tetracycline binding to divalent cations in the gastrointestinal tract) [89].

### Interactions affecting systemic or physiologic dispositions

This type of interaction occurs after the drug molecule or the nutritional constituent has reached the systemic circulation. It might be observed as an alteration in tissue distribution, systemic metabolism, or penetration into a specific tissue. In some clinical situations, the interaction between the drug and the nutrient element may be mediated by cofactors or hormones [84]. One example of this phenomenon is the reduction in the anticoagulant effect of warfarin with a vitamin K-rich diet [90]. Intravenous fat emulsions are supplementary resources of vitamin K and have been correlated with warfarin resistance when used in combination with intravenous nutrition or as delivery system for propofol [91].

### Interactions affecting drug elimination or clearance

This type of interaction influences either hepatic metabolism or renal elimination of the object agents via the involvement of precipitant agents. Numerous pathways may be involved, such as the antagonism, modulation, or diminishment of renal or enterohepatic transportation [84]. High protein diets have been seen to enhance the hepatic elimination of certain drugs like propranolol [92]. The use of aminoglycosides in malnourished patients may be considered as another example of these interactions. Both the volume of distribution and the extracellular fluid compartment may show a significant expansion due to a decrease in the lean tissue mass. Therefore, dosing adjustment of aminoglycosides in malnourished patients must be matched with any alteration in the volume of distribution in order to achieve the appropriate therapeutic drug concentrations [93].

### Nutrient-antiepileptic drugs interactions

Similar to haemoglobin, albumin undergoes an *in vivo *non-enzymatic glycosylation; in fact, approximately 6-10% of the total serum albumin is glycosylated in healthy adult. A group of investigators found that the binding affinities for long chain fatty acid and bilirubin were lower when the glycosylation of albumin was increased [94]. Zimmerman et al. reported that an increase in the concentration of free fatty acids in serum led to an increase in the free fraction of valproic acid [95]. Doucet et al. observed that phenytoin protein binding was significantly decreased in sera from diabetics, but there was no correlation between the percentage of binding and the concentration of glycated albumin [96]. Moreover, Dutkiewicz et al. demonstrated that in hypercholesterolemia and in mixed hyperlipidemia, the free phenytoin concentration was increased. This effect was most likely correlated with the displacement of phenytoin by free fatty acids [97,98]. Accordingly, the free fatty acids from fat emulsions in TPN fluid could displace phenytoin or other drugs from their albumin-binding sites.

Previous studies have shown that drug binding to site II of human serum albumin can be significantly altered by L-tryptophan in amino acid fluids. Because phenytoin can react with site II of human serum albumin [99], these findings are substantially useful for evaluating the pharmacokinetic and pharmacodynamic effects of phenytoin or other therapeutic drugs in patients receiving TPN or a parenteral nutrition solution with amino acids [100]. Furthermore, a decrease in the total phenytoin concentration was reported in a 40-year-old man who started TPN therapy concurrently with phenytoin; the total phenytoin concentration subsequently increased to the pre-TPN concentration following the cessation of TPN therapy [101]. The competitive binding of selected therapeutic drugs to five PN formulas and to human serum *in vitro *has been evaluated using equilibrium dialysis. Phenytoin, phenobarbital, and valproic acid were found to be bound less in the presence of PN than human serum (Table 2), suggesting that the free fraction of these drugs might increase *in vivo *in the presence of these fluids [102].

**Table 2:** Differences in binding of some antiepileptic drugs to parenteral nutrition fluids and to human serum

Drug	Parenteral nutrition formulas	Fluid † (μmol/L)	Serum (μmol/L)	Average Competitive Binding Difference ‡
Carbamazepine	A	16.1	2.1	77%
	B	16.9	1.7	82%
	C	17.8	2.5	75%
	D	16.9	2.1	78%
	E	17.3	2.5	75%
Phenytoin	A	32.4	88.7	-46%
	B	35.0	80.6	-39%
	C	34.1	74.8	-37%
	D	31.7	67.9	-36%
	E	31.7	68.3	-37%
Phenobarbital	A	56.8	75.9	-14%
	B	58.9	74.8	-12%
	C	61.1	77.4	-12%
	D	58.5	77.4	-14%
	E	61.9	78.0	-12%
	A	181.8	1290.2	-75%
Valproic acid	B	194.0	1179.6	-72%
	C	156.7	1061.6	-74%
	D	127.0	993.2	-77%
	E	207.6	998.1	-66%

†A = 4.5% Travasol_/25% dextrose; B = 4.5% Travasol_/10% dextrose; C = 4.5% Travasol_/5% dextrose; D = 2.5% Travasol_/10% dextrose; E = 2.5% Travasol_/5% dextrose. ‡100% × (Concentration in Fluid - Concentration in Serum)/(Concentration in Fluid + Concentration in Serum).

### Alteration of plasma protein binding of antiepileptic drugs

Several factors have been shown to alter the binding of antiepileptic drugs to plasma protein, including hypoalbuminaemia (patients with burns, old age, pregnancy, AIDS etc.) [103,104]; patients with uraemia [105]; drug-drug interactions; displacement of the drug from its plasma protein binding by another drug [106,107]; and patients with chronic liver disease [108].

*In vitro *and *in vivo *studies have been done to observe the displacement of phenytoin from its protein-binding site by antibiotics. The results showed that ceftriaxone, sulfamethoxazole, and nafcillin could all displace phenytoin from serum protein carriers [109].

The effect of albumin and α-1-acid glycoprotein on the binding of therapeutic drugs has been determined by Bailey and Briggs. Their study showed that increasing the concentration of α-1-acid glycoprotein will increase the degree of phenytoin binding by 3-fold, whereas decreasing albumin concentration will decrease the binding by less than 1.2-fold. Phenobarbital and valproic acid exhibited an elevation in their free level when albumin concentration was reduced. Ultimately, conditions like acute inflammation that increase α-1-acid glycoprotein may result in a decrease in the free phenytoin concentration which would not be significantly offset by the decease of albumin levels [110].

### Albumin binding sites

Pharmacokinetics are crucially directed by drug-protein binding in the blood that involve transportation, metabolism and elimination of many pharmaceutical agents. This type of binding can also be a starting place for drug-drug interactions [111-113]. Consequently, it is important to have good knowledge about the type and number of binding sites that a certain drug will interact with on a given protein because this information allows us to estimate how this agent will be influenced by other substances.

**Figure 1:**
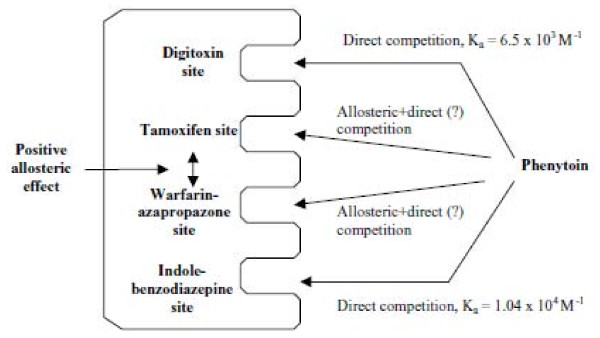
**Proposed model for phenytoin binding to HSA.** The data in this report is consistent with a model in which phenytoin has direct binding at both the warfarin-azapropazone and tamoxifen sites, although it is possible that the competition noted in this study could have been due to negative plus positive allosteric effects between this drug and *R*-warfarin or *cis*-clomiphene. From Chen et al. (2004) [99] with permission of the authors and the publisher.

Albumin is considered to be the most important plasma protein that is involved in such interactions. The warfarin-azapropazone site (Sudlow Site I) and the indole-benzodiazepine site (Sudlow Site II) are the two major albumin binding sites for drugs [114]. Additionally, there are several minor binding sites on albumin for various drugs such as digitoxin and tamoxifen [115,116]. High performance affinity chromatography was used to study the binding of phenytoin to albumin. This study found that phenytoin can interact with albumin at the warfarin, benzodiazepine, tamoxifen and digitoxin sites of this protein (Figure 1) [99]. Dasgupta and Timmerman have studied the *in vitro *phenytoin displacement from protein binding by some non-steroidal anti-inflammatory drugs in both normal and uremic sera. The results illustrated that free phenytoin levels were significantly increased with ibuprofen in both normal and uremic sera [117]. Due to the re-equilibrium phenomena, the in vivo situation might be slightly different. The free phenytoin concentration may not change; it is the free fraction that changes. Ibuprofen-phenytoin interactions can be predicted from their albumin binding sites. Kragh-Hansen et al. showed that ibuprofen is a selective drug probe for site II of human serum albumin [118].

### Monitoring free drug concentrations

Many drugs bind to various plasma proteins (albumin & α-1-acid glycoprotein). This protein binding can be low, moderate or high (>80%). Some drugs, such as ethosuximide and lithium, don't bind to any plasma proteins [119]. Protein binding of most monitored therapeutic drugs is shown in Table 3[108]. Reversible equilibrium and law of mass action govern the existence of drugs in the peripheral circulation as free (unbound) and bound to proteins. Only free drug can produce the pharmacological action by crossing the plasma membrane and binding with the receptors [119]. In addition, the free drug component can freely cross blood-CSF or blood brain barriers [120]. In general, drugs that highly bind plasma proteins are the most important candidates for monitoring of free drug concentrations because variation in protein binding may produce a clinically noteworthy effect in altering free drug concentrations. Newer drugs such as mycophenolic acid, mofetil and certain protease inhibitors are considered to be good candidates for monitoring free drug concentrations. However, free drug concentrations of the old generation of antiepileptic drugs such as phenytoin, carbamazepine and valproic acid are still the most requested by clinicians [108].

**Table 3:** Protein binding of antiepileptic drugs

Drug	Protein binding	Candidate for free drug
**Old generation AED**		
Phenobarbital	40%	No
Phenytoin	90%	Yes
Carbamazepine	80%	Yes
Valproic acid	90-95%	Yes
Primidone	15%	No
Ethosuximide	0%	No
**New generation AED**		
Eslicarbazepine acetate	30	No
Felbamate	25	No
Gabapentin	0	No
Lacosamide	15	No
Lamotrigine	55	No
Levetiracetam	0	No
Oxcarbazepine	40	No
Pregabalin	0	No
Rufinamide	30	No
Stiripentol	99	Yes
Tiagabine	96	Yes
Topiramate	15	No
Vigabatrin	0	No
Zonisamide	50	No

### Old generation antiepileptic drugs

Traditional antiepileptic drugs such as valproic acid, carbamazepine and phenytoin are highly bound to protein. The clinical usefulness of monitoring free concentrations of those drugs has already been studied and documented. Furthermore, the external survey specimens of the College of American Pathologists have also listed the free anticonvulsant concentrations, and assay kits for measurement of free concentrations of these drugs are commercially available [108].

The plasma protein binding of valproic acid ranges from 90 to 95% and occurs mainly with albumin [121]. Because of the saturable binding phenomenon, valproic acid shows vacillation in its protein binding within the therapeutic range (50-100 μg/ml), leading to fluctuation of the free fraction from 10 to 50% [122]. Moreover, several studies revealed that there is no correlation between therapeutic response to valproic acid and its total serum concentration [123,124]. Meanwhile, free valproic acid concentration has been demonstrated to have clinical benefit in the management of epilepsy and the evasion of unwanted side effects [125]. The significance of monitoring free valproic acid in a heart transplant recipient with hypoalbuminaemia has been shown in a case report by Haroldson et al. After the dosing adjustment of valproic acid was based on free concentration rather than total concentration, the patient improved and was finally discharged from the hospital [126]. A case of neurotoxicity has been reported by Gidal et al., in which a hypoalbuminaemic patient exhibited a drastic increase in the plasma free valproic acid concentration with a relatively normal total concentration [127].

In addition, interindividual variation of total valproic acid plasma concentration and its tendency to underrate the effect of poor compliance was observed, but great advantages can be derived by depending on free valproic acid concentration instead of total concentration in therapeutic drug monitoring [128].

Phenytoin is a narrow therapeutic index anticonvulsant drug that appears to follow the Michaelis-Menten pharmacokinetics [129,130]. Approximately 90% of plasma phenytoin is bound to serum albumin [129]. Therapeutically, phenytoin plasma concentrations range from 10-20 μg/ml, which is representative of the total phenytoin concentration [131,132]. Free phenytoin was reported by Soldin to be the most requested measurement of free drug level by clinicians. It was found to be more reliable for the prediction of phenytoin therapy than the total concentration because only free molecules can pass through the blood brain barrier and produce the therapeutic effects [133].

In a retrospective study conducted in Japan by Iwamoto et al. [134], they investigated the relationship between the total or free phenytoin concentration and clinical response, as well as the factors that influence free phenytoin concentration. The study demonstrated that free phenytoin concentrations were poorly correlated with total phenytoin concentrations in both monotherapy and multidrug therapy patients. Free phenytoin concentrations were found to be strongly correlated with the [total phenytoin]/[serum albumin] ratio in patients receiving monotherapy. However, no significant correlation was seen between the free phenytoin concentrations and the [total phenytoin]/[serum albumin] ratio in patients receiving multidrug therapy.

Patients were classified according to their clinical response into the following three groups: complete response, partial response and no response. There was no significant difference among the three groups in the total and free phenytoin concentration in patients receiving multidrug therapy. While the total phenytoin concentration tended to be higher in the complete response group than in the partial response group in monotherapy patients, the difference was not statistically significant. On the other hand, free phenytoin concentrations were significantly higher in the complete response group than in the partial response group of patients receiving monotherapy.

Both the mean serum albumin and the mean creatinine clearance were significantly lower in patients with a high free fraction of phenytoin. However, there was no significant correlation between creatinine clearance and serum albumin levels. Therefore, the effect of creatinine clearance levels on phenytoin protein binding is unrelated to the serum albumin levels. At the same time, the mean age was significantly higher in patients with a high free fraction of phenytoin than in those with a normal or low free fraction. Moreover, Banh et al. have observed that most phenytoin toxicities developed in patients with normal or low total phenytoin concentrations, but with an elevated free concentration of phenytoin [135].

### New generation antiepileptic drugs

In the last two decades, several new antiepileptic drugs have been commercially introduced in the United States and/or Europe. These drugs are tiagabine, eslicarbazepine acetate, stiripentol, felbamate, rufinamide, gabapentin, pregabalin, lacosamide, oxcarbazepine, lamotrigine, topiramate, levetiracetam, vigabatrin and zonisamide. It is well documented that these newer agents exhibit better clinical characteristic (i.e. less sever adverse effect and broader therapeutic range) than the older antiepileptic drugs. Similar to the traditional antiepileptics, some of the newer agents may also be indicated for other clinical situations such as chronic pain syndromes and bipolar disorder [136-138]. Most of the newer agents show little binding to plasma protein (Table 3) [108,139]. However, some of the new antiepileptic drugs do not have any affinity to plasma protein like gabapentin, levetiracetam, pregabalin, and vigabatrin. Only two of the newer antiepileptic drugs (i.e. tiagabine and stiripentol) are considered highly plasma protein binding [139].

Tiagabine has well documented in several observational studies and case reports to cause non-convulsive status epilepticus [140-142]. Presently, tiagabine is rarely used in the United States and Europe [136]. Tiagabine is highly plasma protein binding drug (> 96%). Displacement of tiagabine from its plasma protein binding sites may occur by valproic acid, causing elevation in the free tiagabine concentration [143]. Clinically, a reference range of 20-200 ng/ml has been designed for tiagabine [144]. Numerous analytical approaches are available for the measurement of tiagabine plasma concentration such as HPLC and GC/MS [145,146]. However, with some assays not attaining a low enough limit of sensitivity. The measurement of free tiagabine concentration has been a challenging problem [147].

Stiripentol is one of the new antiepileptic drugs that is highly protein bound (>99%). It was approved in Europe in 2001 [139]. Even though, stiripentol serum concentration of 4-22 mg/L correlates with control of absence seizure in childhood. A clear therapeutic range for stiripentol is not well-proposed [148]. Stiripentol typifies complicated pharmacokinetic characteristics (extensive metabolism, non-linear pharmacokinetics, and highly plasma protein binding) similar to those of the old antiepileptic drugs phenytoin [149]. Attention should be given when stiripentol is used with other antiepileptic drugs like carbamazepine, clobazam, phenobarbital, phenytoin, and valproic acid not only for the displacement from the plasma protein, but for the inhibitory effect of stiripentol on the liver metabolizing enzyme of these drugs and/or metabolites as well [150,151].

### Estimation of free phenytoin levels by applying mathematical equations

Several equations were developed to estimate the free phenytoin concentrations from the measured total phenytoin concentrations and albumin concentrations. Beck et al. investigated the reliability of different equations for predicting free phenytoin concentrations. The authors compared the estimated free concentrations with the measured values. The following three equations were involved in this study: the Gugler method, the Sheiner-Tozer equation and the Sheiner-Tozer nomogram. The results show that all three equations for estimating free phenytoin concentrations were unreliable and carried a lot of bias in predicting free phenytoin concentrations [152]. Furthermore, use of the Sheiner-Tozer equation was not dependable for directing therapy in critically ill paediatric patients [153]. Accordingly, direct measurement of free phenytoin concentrations is preferred to the prediction of free phenytoin concentrations using mathematical equations.

### Assay techniques for free drug concentrations

The most commonly used method for measuring the free concentration of drugs is equilibrium dialysis, in which an appropriate buffer and a sample (containing drug and protein) are separated by a semipermeable membrane. At equilibrium, free drug concentration is the same in the both chambers (in the sample compartment and in the buffer compartment). After dialysis, the total drug concentration is lower than in the early sample (due to dilution). This may lead to serious complexities in the process of result interpretation, especially when the protein binding depends on the drug concentration (i.e., nonlinear binding) [154-156]. Equilibrium dialysis is not practical for clinical implication; therefore, ultrafiltration has become the most preferable technique for monitoring free drug concentrations in clinical laboratories. A small volume of serum (0.8-1.0 ml) is centrifuged for 15-20 minutes to get the ultrafiltrates. Then the concentrations of free drug are determined in the protein free ultrafiltrates [108]. The length of centrifugation time is essential for determining free drug concentration. Liu et al. found that there is a considerable difference between free valproic acid concentrations in the ultrafiltrate when samples were centrifuged samples for 5 minutes compared to 10 minutes or 20 minutes. The authors observed that centrifugation for 5 minutes may not be enough time to yield the actual free concentrations. Therefore, it is recommended to centrifuge the specimens for at least 15 minutes [157]. McMillin et al. reported that the volume of ultrafiltrates were directly proportional to the centrifugation time and were inversely proportional to albumin serum concentrations. Although ultrafiltrate volume was substantially increased with increasing centrifugation time, the free phenytoin concentration did not change proportionately, meaning that equilibrium was sustained between the ultrafiltrate and serum held in the ultrafiltration device [158]. Determination of free phenytoin, valproic acid and carbamazepine concentrations in the ultrafiltrates can be achieved by immunoassays [108]. Free phenytoin concentration in the protein free ultrafiltrate can also be determined by liquid chromatography combined with tandem mass spectrometry [159].

### Critical analysis of lack of evidence

This review came in touch with the importance and significance of careful monitoring of free antiepileptic drugs concentration in patients receiving PN. However, a clear shortage of formal research evidence was found. The wrapping up of this article was based on several anecdotes and case reports. Subsequently, a careful description of the number and the type of studies were not presented. According to the evidence-based approach [160], case report was ranked as the lowest source of evidence to influence the pattern of the decision makers about the therapeutic alternatives. Mostly because these case reports and anecdotes are collected in an unsystematic technique. So that, the outcome of these anecdotes cannot reaches to the level of valid generalizable information [161]. Clinical practice guidelines are developed in systematic approaches to help practitioners and patients decisions about the suitable health care for certain clinical situations [162]. In the same time, these guidelines don't behave as replacement for the role of practitioners and patients in designing the clinical judgment, but it is considered as integrating tools, not rules. So that, the potency of anecdotes can be realized in complex clinical decision phenomena, when there is a limited systematic review of the literature [161].

Enkin and Jadad [163] explained the importance and the influence of anecdotes in making clinical decision, and Jadad [164] previously revealed the strength of the case report within the evidence-based paradigm. When the point is going in the same trend, anecdotes and formal research evidence may support each other, and can have an input on practice far better than could be attained by either alone. Anecdotes can present research findings in more meaningful approaches. It can personalize, illustrate, and market the formal research findings [163,165]. When the research evidence reveals a sense of balance between the beneficial and adverse effects of a treatment, or when it proposes that the treatment alternatives are approximately equivalent, anecdotal information besides the value and the preference of the decision maker can play a significant role to reach the final decision.

In some occasions, the evidence from formal research is not obtainable. In such scenario the decision maker must choose whether or not there is a necessity to explore for such evidence. Predominantly, this investigation is not ensured, because the cost or the efforts need for further exploration might not be justified, especially when the decisions to be made are too measly or unimportant. In this case anecdotes offer the most excellent and only information on which the decision can be made. Moreover, this anecdotal information will enhance the motivation of the researchers to perform the necessary formal research, and will serve as a basis to provide the hypotheses for more definitive studies. Sometimes, it might be difficult to implement formal studies, because the clinical problem may be uncommon or the required studies not are feasible or attractive to financiers [163].

Depending on what have been mentioned above, the authors of this review used several case reports and anecdotes as powerful tools and vehicles to motivate the researchers to perform the necessary formal research evidence.

## Conclusions

There was no evidence supporting the existence of parenteral nutrition-antiepileptic drugs interaction due to the lack of prospective, randomized, controlled trials. However, alteration in the drug-free fraction resulting from coadministration of PN fluids may be clinically important and may necessitate scrupulous reassessment of drug dosages in patients receiving these courses of therapies. This reassessment may be particularly imperative in patients with certain clinical situations in which there are reductions in the levels of binding proteins (e.g., burn patients). Most patients who receive PN may also take some therapeutic drugs, leading to potential interactions that may contribute to adverse events. Surprisingly, there are very few published studies on the interaction of PN fluids with therapeutic drugs, and most of them do not focus on protein binding [102,166-169].

## Competing interests

None of the authors have accepted or received any honoraria or promise of future benefits or any other financial support that may inappropriately influence this work or may lead to some source of bias in designing, writing and finalizing this review article.

## Authors' contributions

All authors have made substantial contributions to the conception of the study, drafting the article, and final approval of the version to be submitted. MS and MB conceived and designed the study. MS and AA did the electronic search for the relevant articles and drafted the manuscript. MB revised and edited the manuscript. MS and AA prepared the manuscript for publication. All authors have read and approved the final submitted manuscript.
